# Evaluating Vaginal Microbiome Effects on Antiretroviral Exposure in the Female Genital Tract Using Pharmacometric and Machine Learning Approaches

**DOI:** 10.1002/cpt.70499

**Published:** 2026-10-03

**Authors:** Diqin Yan, Lindsey B. Collins, Christopher Staley, Flavia Kiweewa Matovu, Pamala Jacobson, Melanie Nicol, Shen Cheng

**Affiliations:** ^1^ Department of Experimental and Clinical Pharmacology, College of Pharmacy University of Minnesota Minneapolis Minnesota USA; ^2^ Division of Basic and Translational Research, Department of Surgery, Medical School University of Minnesota Minneapolis Minnesota USA; ^3^ Makerere University‐Johns Hopkins University Research Collaboration Kampala Uganda; ^4^ College of Health Sciences Makerere University Kampala Uganda

## Abstract

Antiretroviral pre‐exposure prophylaxis (PrEP) is less effective in women than expected, partly because drug exposure within the female genital tract is variable and may be influenced by the vaginal microbiome. We developed an integrated pharmacometrics and machine learning framework to evaluate associations between vaginal microbiome composition and cervical antiretroviral exposure in women with human immunodeficiency virus (HIV) receiving tenofovir (TFV)‐, lamivudine (3TC)‐, or emtricitabine (FTC)‐containing regimens. Pharmacokinetic and microbiome data were obtained from two single‐center studies in Uganda and the United States. Published population pharmacokinetic models were used to estimate individual parameters by maximum a posteriori Bayesian estimation and to simulate plasma and cervical tissue exposure metrics under sexual‐activity‐driven dosing that were not studied in these trials. Microbiome diversity was assessed using alpha and beta diversity analyses. Associations between microbial genera and cervical drug exposure were evaluated using correlation analysis, differential abundance testing, and three machine learning approaches: LASSO, random forest, and XGBoost. Overall vaginal microbiome diversity was not associated with cervical exposure to TFV, 3TC, or FTC. In contrast, specific taxa‐level associations with cervical drug exposure were identified. For TFV, *Gemella* was positively associated with cervical TFV‐diphosphate exposure, whereas *Megasphaera* and *Falsiporphyromonas* were negatively associated across multiple models. For 3TC, *Dialister, Prevotella, Atopobium, Streptobacillus*, and *Gardnerella* were repeatedly identified. For FTC, *Bifidobacteriaceae* was consistently selected, with *Lachnospiraceae* and *Prevotellaceae* also implicated. This pharmacometrics‐machine learning framework identified specific vaginal taxa associated with cervical antiretroviral exposure, supporting the future development of microbiome‐based biomarkers and precision PrEP strategies for women.

**Study Highlights**

**WHAT IS THE CURRENT KNOWLEDGE ON THE TOPIC?**

Antiretroviral pre‐exposure prophylaxis (PrEP) is less effective in women than expected, partly because drug exposure at the female genital tract is variable and may be influenced by the vaginal microbiome. Prior studies have suggested that bacterial vaginosis‐associated taxa can reduce mucosal tenofovir (TFV) exposure and potentially compromise HIV protection. However, defining associations between specific bacterial genera and antiretroviral exposure remains challenging due to the dynamic nature of drug exposure and the high dimensionality of microbiome data.

**WHAT QUESTION DID THIS STUDY ADDRESS?**

This study asked whether specific vaginal bacterial genera are associated with variability in cervical antiretroviral exposure in women receiving tenofovir, lamivudine (3TC), or emtricitabine (FTC)‐containing regimens.

**WHAT DOES THIS STUDY ADD TO OUR KNOWLEDGE?**

This study showed that overall microbiome diversity was not associated with cervical antiretroviral exposure, whereas specific taxa demonstrated significant associations: *Gemella, Megasphaera*, and *Falsiporphyromonas* for TFV; *Dialister, Prevotella, Atopobium, Streptobacillus*, and *Gardnerella* for 3TC; and *Bifidobacteriaceae, Lachnospiraceae*, and *Prevotellaceae* for FTC.

**HOW MIGHT THIS CHANGE CLINICAL PHARMACOLOGY OR TRANSLATIONAL SCIENCE?**

This work provides an integrated framework that combines pharmacometric modeling to characterize dynamic drug exposure with machine learning to analyze high‐dimensional microbiome data. The approach may support future development of practical microbiome‐based biomarkers, inform model‐informed precision dosing strategies, and help optimize PrEP effectiveness in women.

Approximately 40 million people worldwide are living with human immunodeficiency virus (HIV), and over half of them are female.^1^ Women, particularly those in sub‐Saharan Africa, face disproportionately high infection rates, with young women experiencing HIV incidence rates more than three times higher than young men.^2^ These disparities underscore an urgent need for effective prevention strategies tailored to women. Oral antiretroviral pre‐exposure prophylaxis (PrEP) using a fixed‐dose combination of tenofovir disoproxil fumarate and emtricitabine (TDF/FTC) has demonstrated high efficacy in men who have sex with men^3^; however, clinical trials in heterosexual women have reported inconsistent efficacy.^4^, ^5^ While suboptimal adherence contributes to variability in treatment outcomes, biological factors, such as differential drug penetration into mucosal tissues and the influence of the vaginal microbiome, likely also play pivotal roles.^6^, ^7^ Understanding these female tissue‐specific biological determinants is critical for the improvement of PrEP effectiveness in women.

Among factors contributing to variability in PrEP treatment outcomes, the vaginal microbiome has emerged as a critical factor, influencing mucosal health, and potentially modulating PrEP pharmacology.^7^, ^8^, ^9^ Bacterial vaginosis, a clinical manifestation of vaginal dysbiosis, is associated with poorer outcomes, likely due to its effect in reducing tenofovir (TFV) concentrations in vaginal tissues.^10^ Notably, *Gardnerella vaginalis*, a dominant bacterial vaginosis‐associated species, has been shown to metabolize TFV *in vitro*, thereby lowering local active drug concentrations and potentially compromising HIV protection.^10^ Collectively, these microbiome‐drug interactions within female genital tracts provide mechanistic insights into reduced PrEP efficacy observed in women.

Despite the recognized impact of bacterial vaginosis on antiretroviral drug exposures, elucidating the associations between specific bacterial genera and drug concentrations remains challenging due to the dynamic nature of drug exposure (i.e., pharmacokinetics, PK), and complexity of microbial communities along with their interactions with host factors. First, mucosal antiretroviral concentrations are dynamic, and a single measurement (“snapshot”) does not reflect the complexity of exposure over time.^11^ Second, vaginal microbiome is high‐dimensional, sparse, and compositional, creating analytical challenges such as overfitting and limited generalizability.^12^, ^13^ As a result, existing methods lack the capacity to jointly model dynamic PK data and high‐dimensional microbiome features in a unified, quantitative framework.

To address these challenges, we developed an innovative framework that integrates pharmacometric (PMx) modeling with machine learning (ML) analysis. The PMx captures the dynamic drug exposures over time, while ML enables efficient exploration of complex multivariate associations to identify bacterial taxa correlated with variations in drug exposure.^12^, ^14^ Using this integrated PMx‐ML approach, we aim to identify microbiome genera impacting antiretroviral drug exposure in the female genital tract.

## MATERIALS AND METHODS

### Data

Data were obtained from two single‐center, single‐visit PK studies (**Table** **1**).^15^, ^16^ The Uganda and US studies enrolled 50 and 21 women living with HIV, respectively, who were stable on an oral antiretroviral regimen and virally suppressed for ≥ 6 months. In the Ugandan cohort, all participants received TDF/lamivudine (3TC) (300/300 mg once daily) in combination with efavirenz 600 mg (*n* = 49) or boosted atazanavir 300 mg (*n* = 1).^15^ In the US cohort, participants received either TDF/FTC (300/200 mg once daily) or tenofovir alafenamide (TAF)/FTC (25/200 mg once daily), in combination with heterogeneous third anchor drugs (**Table** **1**).^16^ Plasma parent drug concentrations, cervical intracellular metabolite concentrations, and vaginal microbiome profiles were collected at a single visit. The datasets were analyzed separately by drug and cohort: TFV and 3TC were evaluated using only the Ugandan cohort, while FTC was evaluated using only the US cohort. The US cohort included participants receiving either TDF or TAF, which have distinct systemic and female genital tract PK profiles.^17^, ^18^ Therefore, the reference TFV model developed using data from TDF‐based regimens only was not applied to participants receiving TAF. After excluding these participants, too few US participants receiving TDF remained to support a meaningful TFV‐specific analysis. Consequently, TFV was evaluated only in the Ugandan cohort. Detailed study design, concentration data collection, and microbiome data processing methods are described in **Supplementary Methods**.

**Table 1:** Summary of available clinical data

Trial #		NCT03377608	NCT03218085
Drug		TDF/3TC: 300/300 mg Q.D.	TDF/FTC: 300/200 mg Q.D. TAF/FTC: 25/200 mg Q.D.
Anchor drug		EFV (*n* = 49); boosted ATV (*n* = 1)	DTG (*n* = 7); EVG (*n* = 6); DRV (*n* = 5); EFV (*n* = 2); ETR (*n* = 1); BIC (*n* = 1); ATV/RTV (*n* = 1); unknown (*n* = 1)^a^
Population		Ugandan women with HIV	American women with HIV
N		50	21
Data availability	PK	TFV/3TC in plasma TFV‐DP/3TC‐TP in cervical tissue dATP/dCTP in cervical tissue	TFV/FTC in plasma TFV‐DP/FTC‐TP in cervical tissue dATP/dCTP in cervical tissue
	Microbiome	16S ribosomal RNA sequencing using vaginal swab samples	16S ribosomal RNA sequencing and shotgun sequencing using vaginal swab samples
	Clinical variables	Age, weight	Age, weight

^a^ Some participants received more than one anchor drug.

Abbreviations: 3TC, lamivudine; 3TC‐TP, lamivudine triphosphate; ATV, atazanavir; BIC, bictegravir; dATP, deoxyadenosine triphosphate; dCTP, deoxycytidine triphosphate; DRV, darunavir; DTG, dolutegravir; EFV, efavirenz; ETR, etravirine; EVG, elvitegravir; FTC, emtricitabine; FTC‐TP, emtricitabine triphosphate; HIV, human immunodeficiency virus; PK, pharmacokinetics; Q.D., once daily; RTV, ritonavir; TAF, tenofovir alafenamide; TDF, tenofovir disoproxil fumarate; TFV, tenofovir; TFV‐DP, tenofovir diphosphate.

The overall study workflow was illustrated in **Figure** **1**. Briefly, an antiretroviral drug PK model was identified in the literature that adequately characterized TFV/FTC PK profiles in both systemic circulation and female genital tract tissues (e.g., vaginal and cervical tissues). Following model extraction and validation of its predictive performance in our data set, individual parameter estimates were obtained from our clinical study data and used to simulate individual drug exposures in both plasma and cervical tissues (blue upper section in **Figure** **1**). These simulated individual exposures were then combined with vaginal microbiome taxa to evaluate (1) the impact of overall microbiome diversity on drug exposures (yellow bottom‐left section); and (2) the impact of individual microbiome genera on drug exposures (red bottom‐right section).

**Figure 1:**
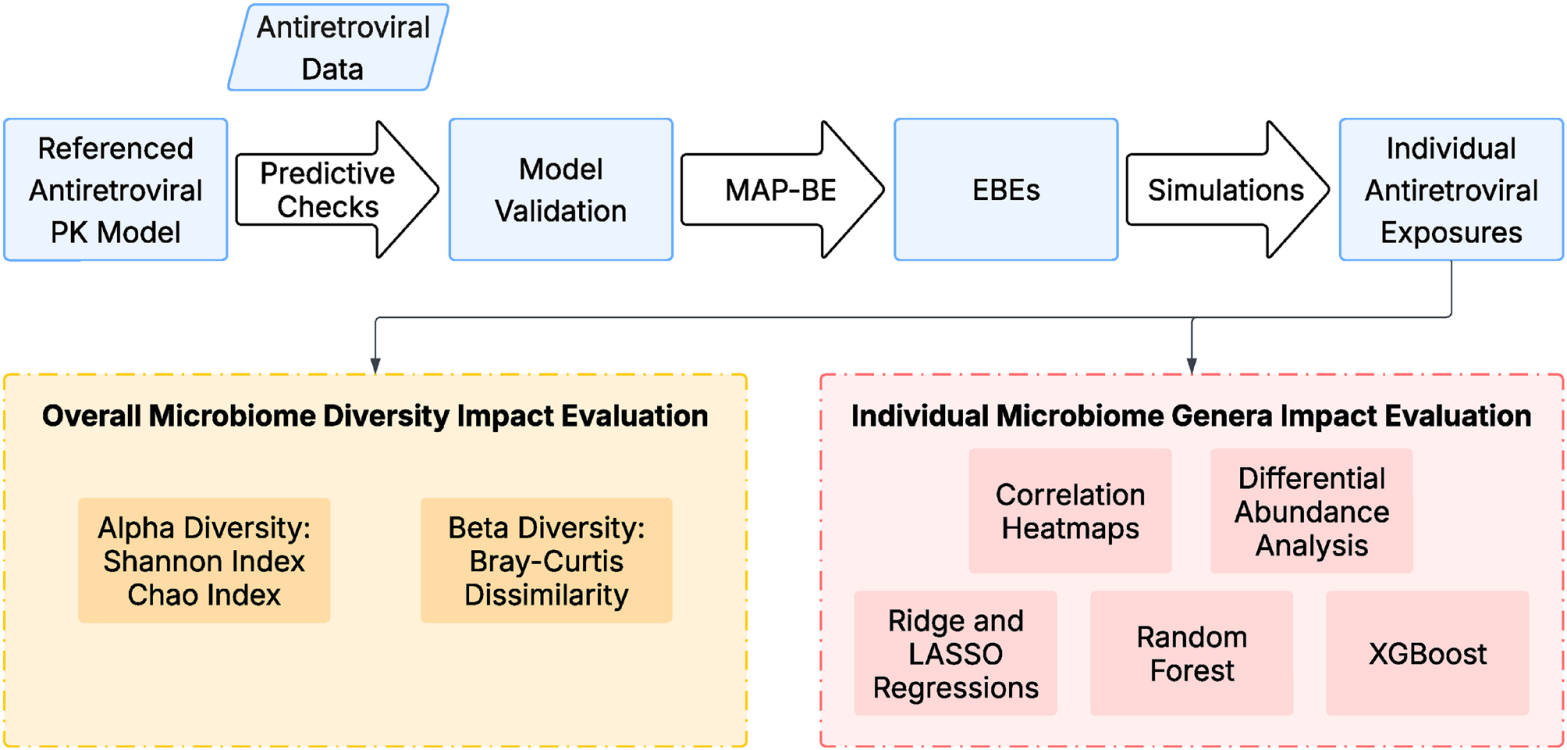
Analysis workflow. MAP‐BE: maximum *a posteriori* Bayesian estimation; EBEs: empirical Bayes estimates; XGBoost: extreme gradient boosting.

### Model‐based estimation and simulation of antiretroviral exposure

Given the single time‐point PK sampling strategies of the clinical studies shown in **Table** **1**, Bayesian estimation of individual PK parameters (i.e., maximum *a posteriori* Bayesian estimation [MAP‐BE]) was performed using published PK models rather than developing new models using our available PK data.

A previously published 16‐compartment population PK model by Leung, E. *et al*. was identified (**Figure** **S1**).^19^ This model was originally developed using data from 23 healthy premenopausal women receiving single oral doses of TDF or FTC. The model incorporates several key components: oral absorption through seven gut transit compartments from the depot, two‐compartment plasma disposition, and distribution to three mucosal tissue sites (vaginal, cervical, and rectal). Each tissue contains both parent drug (TFV/FTC) and active metabolite (tenofovir diphosphate [TFV‐DP]/emtricitabine triphosphate [FTC‐TP]) compartments to describe intracellular phosphorylation. This model was extracted and translated into mrgsolve, an open‐source ordinary differential equation (ODE)‐based simulation engine.^20^ As no population PK model for 3TC was available, the PK model for FTC, and FTC‐TP was applied to characterize 3TC and lamivudine triphosphate (3TC‐TP) disposition, given their similar PK properties.^21^ Model performance was evaluated using visual predictive checks (VPC) with 200 simulation replicates. The previously published population PK model and parameter estimates from Leung et al. were not re‐estimated using our data, which served solely as an external validation dataset. The VPCs therefore represent an out‐of‐sample, simulation‐based predictive check evaluating how well the literature‐based model predicts the concentrations observed in our study.

Individual PK parameters (i.e., empirical Bayes estimates [EBEs]) were derived using MAP‐BE based on the referenced population PK models implemented using the *mapbayr* package (version 0.10.0).^22^ MAP‐BE leverages the *a priori* parameter distributions provided by the population model to estimate EBEs using observed drug concentrations and relevant covariates. Model adequacy was further assessed by examining the distributions of EBEs of each parameter.

The derived EBEs then drove the simulations of fine‐grid concentration‐time (i.e., PK) profiles under two PrEP dosing regimens that were not directly studied in the original clinical trials: (1) event‐driven PrEP (ED‐PrEP), a 2‐1‐1 regimen currently recommended for men who have sex with men, consisting of a double dose of TDF/FTC (or TDF/3TC) administered 2–24 hours before exposure followed by single doses at 24 and 48 hours after the initial dose^23^; and (2) an extended 2‐1‐1‐1‐1‐1 regimen recommended by expert opinion for vaginal protection, followed by three additional daily doses.^24^ Deterministic simulations using derived EBEs were performed using mrgsolve, with details provided in **Supplementary Methods**.

Subsequently, the simulated fine‐grid PK profiles were used to derive the following exposure metrics for both TFV/3TC/FTC in plasma and TFV‐DP/3TC‐TP/FTC‐TP in cervical tissues: area under the concentration‐time curve from 0 to t hours (AUC_0‐t_), peak concentration (C_max_), and average concentration (C_ave_). For TFV‐DP, the fraction of time above predefined clinically relevant efficacy thresholds was calculated: TFV‐DP:dATP ratios of 0.086 (EC_90_ in TZM‐bl cell) and 0.29 (EC_90_ in CD4+ T cell),^25^ and TFV‐DP concentration of 10,700 fmol/mL (cervical explant EC_50_ obtained from cervical *ex‐vivo* model^26^; see Supplemental Methods). Similarly, for FTC‐TP, the fraction of time above established efficacy thresholds was calculated, including FTC‐TP:dCTP ratios of 0.27 (EC_90_ in TZM‐bl cells) and 0.07 (EC_90_ in CD4^+^ T cells).^25^ Under each dosing regimen, the fraction of time above each efficacy threshold was calculated across: (1) 240 hours following the first dose and (2) 24 hours following the final dose.

### Microbial diversity

To analyze variation in vaginal microbiome diversity and composition across drug exposure levels, participants with microbiome data were dichotomized into high‐ and low‐drug exposure groups based on the median cervical tissue AUC from simulation. Alpha diversity within the microbiome samples was assessed using the Shannon index (diversity) and Chao index (richness),^27^ and compared between groups using Wilcoxon rank‐sum tests with statistical significance denoted as *P* < 0.05. Beta diversity, indicating between‐sample microbial community differences, was evaluated using Bray–Curtis dissimilarity distances calculated from relative abundance data,^28^ with group differences tested using permutational multivariate analysis of variance (PERMANOVA). Principal coordinate analysis (PCoA) was performed to visualize clustering patterns associated with drug exposure levels.

### Association analyses between drug exposures and microbiome

Multiple analytical approaches were employed to identify specific microbial genera associated with drug exposure variability. Association analyses were performed separately for TFV, 3TC, and FTC. Plasma drug exposure was represented by the simulated AUC of the plasma parent drug (TFV/3TC/FTC), whereas cervical drug exposure was represented by the simulated AUC of the corresponding intracellular active metabolite (TFV‐DP/3TC‐TP/FTC‐TP). All microbial abundance data underwent centered log‐ratio (CLR) transformation using the *Tjazi* package (version 0.1.0.0) to address compositional constraints before each analysis.^29^, ^30^

#### Correlation Heatmaps

Spearman correlation coefficients were used to characterize the association between microbiome relative abundances and PK exposure metrics. These metrics included plasma parent drug exposure and cervical intracellular metabolite exposure, as well as the fraction of time above efficacy thresholds where applicable. The results were visualized as heatmaps using the *ComplexHeatmap* package (version 2.18.0).^31^

#### Differential abundance analyses

Differential relative abundance analyses were conducted using the *DESeq2* package (version 1.42.1) to identify genera significantly enriched or depleted between high and low cervical drug exposure groups.^32^ Participants were dichotomized based on median cervical tissue AUC values. Taxa present in at least 10% of samples were retained for analysis. The detailed approach is described in **Supplementary Methods**.

#### Machine learning models

Machine learning models including linear regression‐based methods and tree‐based methods were trained separately for TFV and 3TC using the Ugandan cohort and for FTC using the US cohort to identify impactful predictors of cervical drug exposure. Linear regression‐based feature selection was conducted using ridge and LASSO regressions, implemented via the *glmnet* package (version 4.1.9).^33^ The purpose of the analysis is to identify microbial genera associated with cervical drug exposures, while also accounting for systemic drug exposures (corresponding plasma AUC of each drug) and relevant clinical covariates (age and body weight). Tree‐based nonlinear modeling was performed using Random Forest (RF) and eXtreme Gradient Boosting (XGBoost), implemented via the *randomForest* (version 4.7.1.2) and *xgboost* (version 1.7.11.1) packages, to further characterize complex relationships between microbiome genera and cervical drug exposure. The detailed ML approaches are described in **Supplementary Methods**.

### Microbial Co‐occurrence network analysis

Microbial co‐occurrence networks were inferred using the *NetCoMi* package (version 1.2.0) in R, applying the Sparse Correlations for Compositional data (SparCC) method to account for the compositional nature of microbiome data.^34^, ^35^ The detailed description is provided in **Supplementary Methods**.

### Software

All analyses were conducted using R (version 4.3.0, R Foundation for Statistical Computing, Vienna, Austria) with an interface of R studio (version 2023.12.1.402, Posit Software, PBC, Boston, MA, USA) on high performance cluster at the Minnesota Supercomputing Institute.

### Source code

All the source code for the analyses is publicly available on Zenodo (https://doi.org/10.5281/zenodo.22133574).

## RESULTS

### Model validation and simulated exposure

The population PK models developed by *Leung, E. et al*. were successfully compiled in mrgsolve and generally captured TFV, 3TC, and FTC concentration–time profiles observed in both plasma and cervical tissue.^19^ VPC indicated that the majority of observed TFV, 3TC, and FTC plasma concentrations, as well as TFV‐DP, 3TC‐TP, and FTC‐TP cervical levels, fell within the model‐simulated 90% prediction intervals, supporting acceptable predictive performance of PK models (**Figures** **2a**, **S2a**, and **S3a**). Therefore, the model was deemed suitable for deriving EBEs of each antiretroviral drug. The EBEs obtained in relevant tissue compartments using MAP‐BE were approximately normally distributed for TFV, 3TC, and FTC (**Figures** **2b**, **S2b**, and **S3b**) without substantial deviations from the theoretical mean and variance, suggesting the validity of derived EBEs. Notably, while the original population PK models developed by *Leung, E. et al*. incorporated PK data from plasma as well as vaginal, cervical, and rectal tissues, PK data in the present study were available only for plasma and cervical tissues. Consequently, individual parameters associated with vaginal and rectal tissues were expected to shrink toward their population estimates (**Figures** **2b**, **S2b**, and **S3b**).

**Figure 2:**
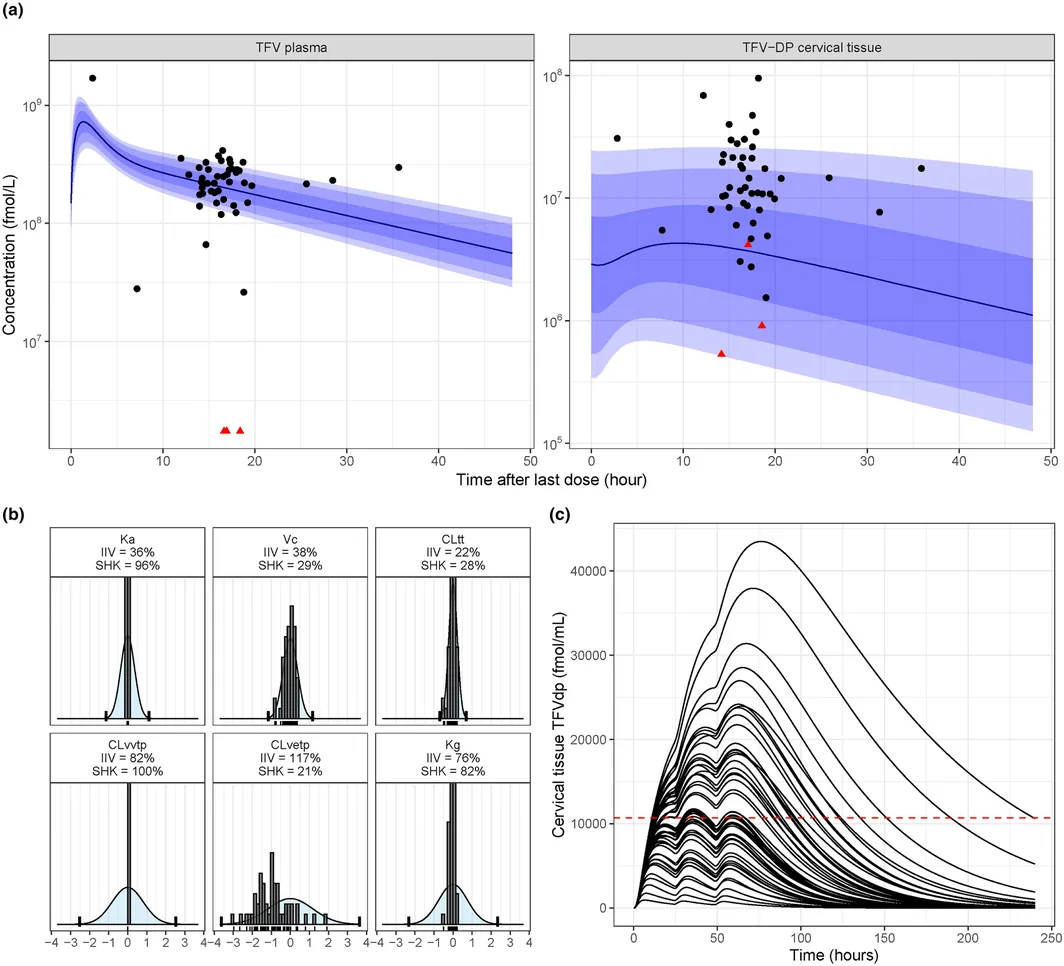
Derive empirical Bayes estimates (EBEs) to drive simulations for TFV. (a) Predictive checks using population PK model developed by Leung, E. *et al*. The points are observed concentrations, with red triangle representing observations below the limit of quantification. The solid line represents the simulated median, and the shaded bands represent the 50%, 80%, and 90% prediction intervals based on 200 simulation replicates. TFV: tenofovir; TFV‐DP: tenofovir diphosphate. (b) Empirical Bayes estimates (EBEs) derived using Maximum A Posterior (MAP) Bayesian method. The histograms represent derived EBE based on the observed plasma and cervical tissue concentration‐time data. Shaded areas represent the theoretical distributions of the parameters. IIV: inter‐individual variability; SHK: shrinkage; Ka: absorption rate constant (/hour); Vc: central volume of distribution (L); CLtt: total drug clearance (L/hour); CLvvtp: clearance rate of metabolite from vaginal compartment (L/hour); Clvetp: clearance rate of metabolite from cervical compartment (L/hour); Kg: gut transit rate (/hour). (c) EBE‐based simulations assuming a dosing on‐demand PrEP regimen (600 mg before sex followed by two 300 mg once daily doses). The red dashed line is the EC50 (10,700 fmol/g) determined in ex vivo cervical explants.

Using these EBEs, simulations were conducted to characterize plasma and cervical exposures under an on‐demand 2‐1‐1 PrEP regimen. Under this regimen, 58% of simulated individuals achieved cervical TFV‐DP concentrations above the putative efficacy threshold (the EC_50_ of ~10,700 fmol/mL in cervical tissue), indicating that TFV‐DP concentrations could reach pharmacologically protective concentrations with 2‐1‐1 dosing (**Figure** **2** **c**). Simulated cervical 3TC‐TP and FTC‐TP concentrations in cervical tissues were also simulated under the 2‐1‐1 regimen, but no cervical tissue concentration efficacy thresholds were identified to contextualize these levels. Drug exposure metric, AUC_0‐240_, was subsequently summarized using the simulated PK profiles of each antiretroviral drug and used for microbiome‐drug‐exposure analyses. Simulations were also conducted under an extended 2‐1‐1‐1‐1‐1 regimen. However, considering the extracted PK models were linear, AUCs derived from simulations under a 2‐1‐1‐1‐1‐1 dosing regimen would be proportional to those under a 2‐1‐1 dosing regimen. Therefore, only AUCs derived from the 2‐1‐1 dosing regimen were used for microbiome‐drug‐exposure analyses. The exposure metrics and the fraction of time above established efficacy thresholds derived from the 2‐1‐1 regimen simulations are summarized in **Tables** **S2 and S3**.

### Microbial diversity

Overall vaginal microbiome diversity did not differ significantly between participants with high vs. low cervical drug exposure for any of the three drugs. The median Shannon and Chao diversity indices (alpha diversity) were similar in high‐ and low‐exposure groups and showed no significant difference for TFV (Wilcoxon *P* > 0.05, **Figure** **S4a,b**), and likewise for 3TC and FTC (**Figures** **S5a,b** **and** **S6a,b**). Similarly, beta diversity analyses indicated no evidence of separation in community structure by drug exposure level. PCoA of Bray‐Curtis distances revealed no clustering of microbiome composition between high and low cervical exposure groups for three drugs (PERMANOVA *P* > 0.05; **Figures** **S4c**, **S5c**, **and** **S6c**). These findings suggest that cervical exposure to TFV, 3TC, or FTC is not associated with overall vaginal microbiome diversity.

### Exploratory correlation Heatmap analysis of microbial genera and cervical exposure

Although overall diversity and community structure appeared unrelated to cervical drug concentrations, several bacterial genera showed notable correlations with drug exposure metrics. Reduced correlation heatmaps show the associations between CLR‐transformed relative abundances of predominant genera and simulated drug exposure metrics (**Figure** **3** **a**, **S7a**, and **S8a**). Higher cervical TFV‐DP AUC was positively correlated with *Mycoplasma* relative abundance and negatively correlated with *Falsiporphyromonas* relative abundance (**Figure** **3** **a**). Cervical 3TC‐TP exposure showed a positive correlation with *Lactobacillus* relative abundance and a negative correlation with *Streptobacillus* (**Figure** **S7a**). Cervical FTC‐TP exposure was positively correlated with *Bifidobacteriaceae* (not further classified to genus) and *Lactobacillus* relative abundance and negatively correlated with *Lachnospiraceae* (not further classified) relative abundance (**Figure** **S8a**).

**Figure 3:**
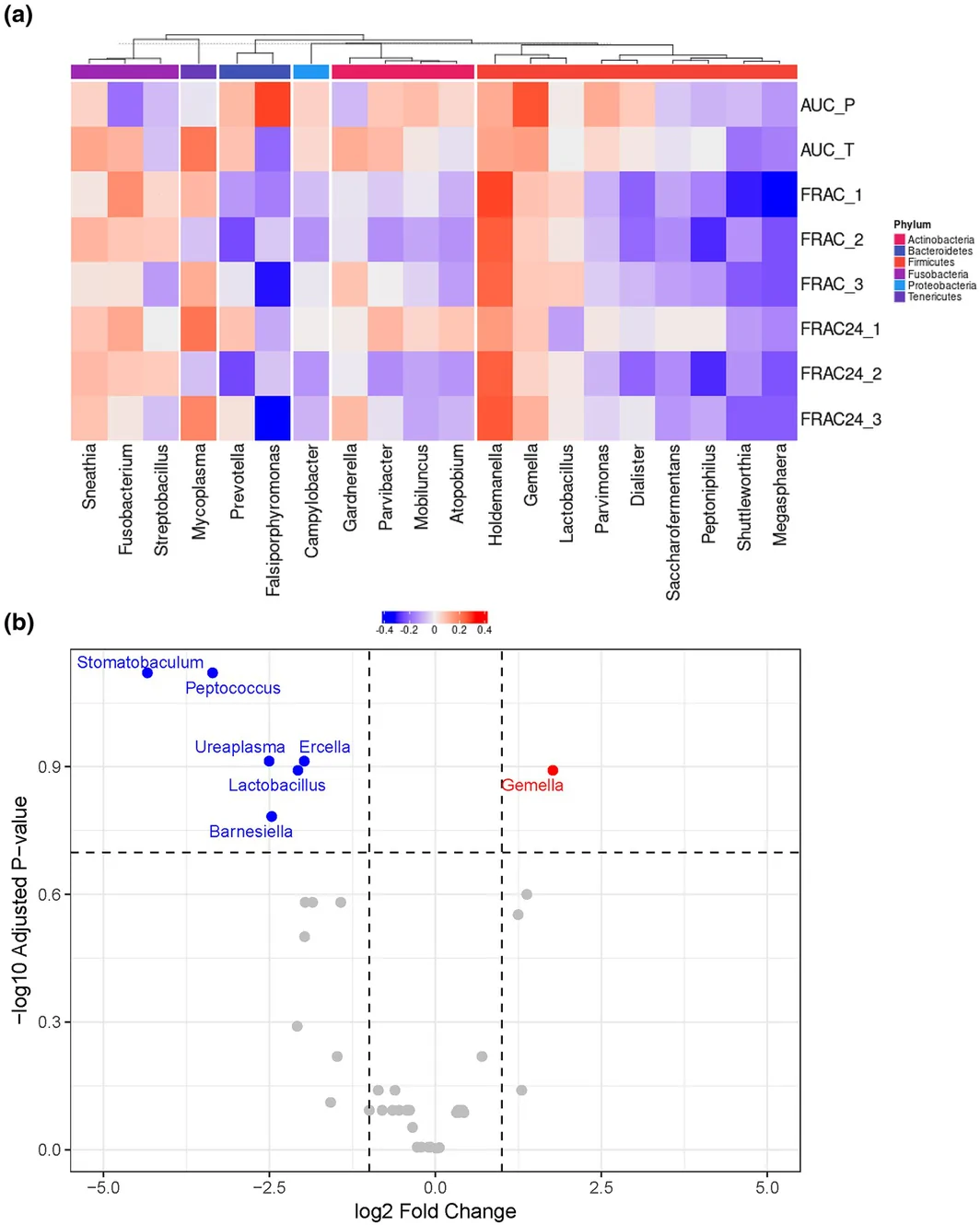
Associations between vaginal microbiome composition and cervical TFV exposure. (a) Heatmap showing the associations between CLR‐transformed microbiome relative abundance and simulated drug exposure metrics. Top color bar represents the microbiome phylum. AUC_P: plasma TFV AUC; AUC_T: cervical tissue TFV‐DP AUC; FRAC_1: fraction of time above threshold 1 (TFV‐DP:dATP = 0.086, TZM‐bl cell)^25^; FRAC_2: fraction of time above threshold 2 (TFV‐DP:dATP = 0.29, CD4+ T cell)^25^; FRAC_3: fraction of time above threshold 3 (10,700 fmol/g, cervical explants EC_50_); FRAC24_1: fraction of time above threshold 1 (TFV‐DP:dATP = 0.086, TZM‐bl cell) within 24 hours post‐dose; FRAC24_2: fraction of time above threshold 2 (TFV‐DP:dATP = 0.29, CD4+ T cell) within 24 hours post‐dose; FRAC24_3: fraction of time above threshold 3 (10,700 fmol/g, cervical explants) within 24 hours post‐dose. (b) Volcano plot showing differential abundance analysis comparing high versus low cervical TFV‐DP AUC groups, dichotomized using the median simulated cervical TFV‐DP AUC. The horizontal dashed line indicates the false discovery rate (FDR) threshold (0.2), and vertical dashed lines denote log_2_ fold change cutoffs. Genera meeting the FDR threshold are highlighted and labeled, with red indicating enrichment in the high‐exposure group and blue indicating enrichment in the low‐exposure group.

### Exploratory differential abundance analysis between high and low‐exposure groups

Few taxa were identified as significantly different in relative abundance when comparing high vs. low‐exposure groups, and this pattern differed by drug. For TFV, differential abundance testing between high and low cervical TFV‐DP AUC (using a lenient FDR < 0.2 threshold given the exploratory analysis) identified several genera with significant differences. *Gemella* was enriched in the high exposure group, whereas *Stomatobaculum*, *Peptococcus*, *Ureaplasma*, *Ercella*, *Lactobacillus*, and *Barnesiella* were more abundant in the low‐exposure group (**Figure** **3** **b**). In contrast, no genus showed a statistically significant difference in relative abundance between the high and low 3TC‐TP cervical exposure groups (**Figure** **S7b**). For FTC, *Lachnospiraceae* was enriched in the low exposure group (**Figure** **S8b**).

### Machine learning with regularized regression modeling: Ridge and LASSO

Both ridge and LASSO regression models were applied to identify microbiome predictors that could predict cervical drug concentrations. While ridge models retain all variables, LASSO models performed covariate screening, which only retain key predictors. In the LASSO model for TFV, in addition to plasma TFV AUC and body weight, *Gemella* was identified as a positive predictor of cervical TFV‐DP AUC, while *Megasphaera, Streptobacillus*, and *Falsiporphyromonas* emerged as negative predictors (**Figure** **4** **a,b**). In the LASSO model for 3TC, alongside plasma 3TC AUC and age, *Lactobacillus*, and *Gardnerella* were identified as positive predictors, whereas *Streptobacillus* was identified as a negative predictor of cervical 3TC‐TP AUC (**Figure** **S9a,b**). In the LASSO model for FTC, age was retained among the top six predictors. Among microbial predictors, *Prevotella* and *Megasphaera* were positively associated with cervical FTC‐TP AUC, whereas *Bifidobacteriaceae*, *Dialister*, and *Mobiluncus* were identified as negative predictors (**Figure** **S10a,b**).

**Figure 4:**
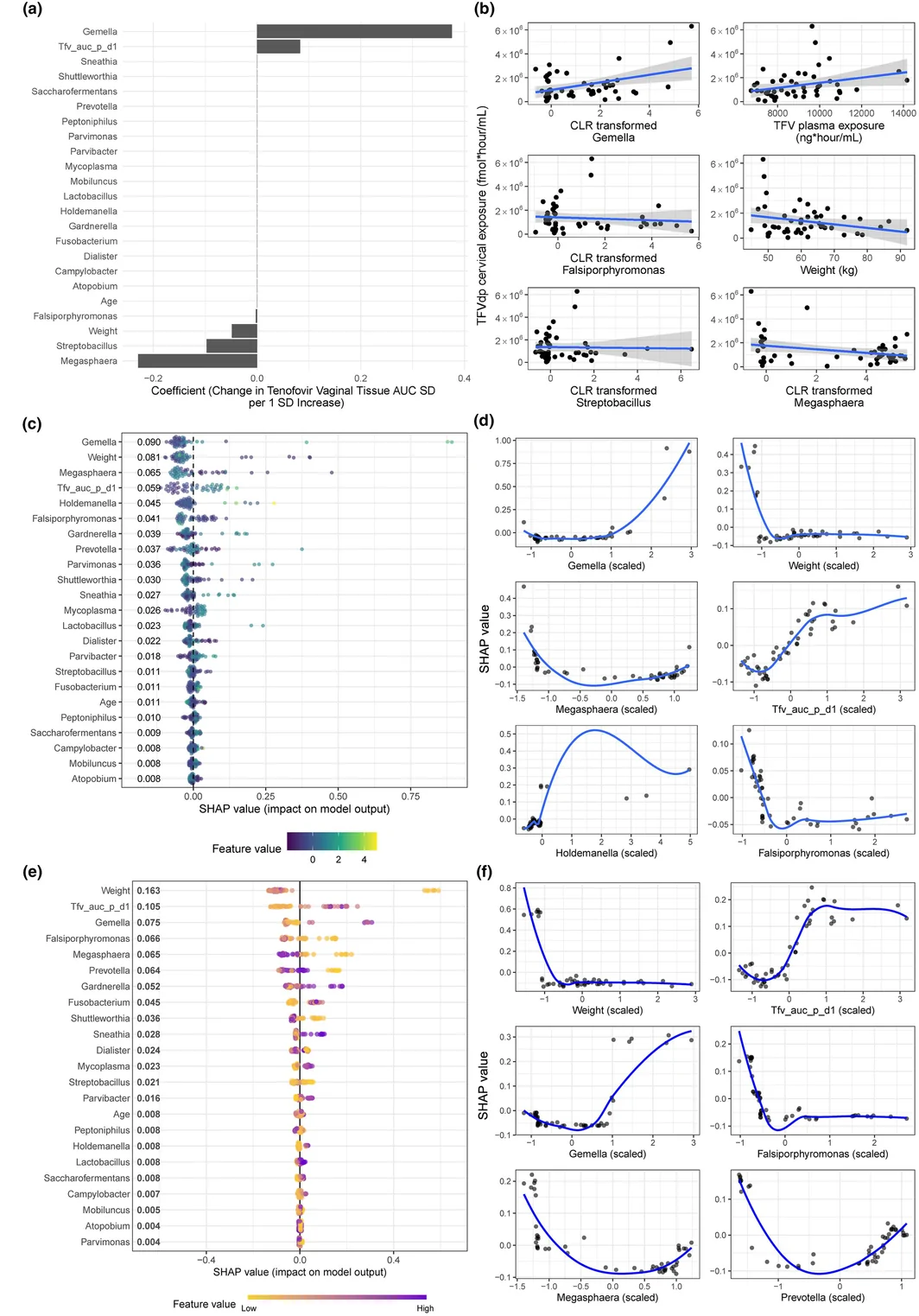
Machine learning‐based analyses of predictors associated with TFV‐DP cervical exposure. (a) Standardized coefficients from the LASSO regression model for TFV‐DP cervical tissue AUC. (b) Scatterplots of TFV‐DP cervical tissue AUC vs. LASSO‐screened predictors. (c) SHAP summary plot from the random forest model, ranking predictors by their overall contribution to model predictions. (d) SHAP dependence plots from the random forest model for top‐ranked predictors. (e) SHAP summary plot from XGBoost model, ranking predictors by their overall contribution to model predictions. (f) SHAP dependence plots from XGBoost model for top‐ranked predictors. For SHAP summary plots, each point represents one observation for a predictor. The x‐axis shows the SHAP value, indicating the direction and magnitude of that predictor's contribution to the predicted TFV‐DP cervical tissue AUC; positive values increase the prediction, whereas negative values decrease it. Colors indicate relative predictor values from low to high, as shown in the color scale. Tfv_auc_p_d1: TFV plasma AUC under 2‐1‐1 dosing regimen.

### Machine learning with tree‐based nonlinear modeling: Random Forest and XGBoost

Random forest models and XGBoost identified several key microbial and clinical predictors of cervical drug exposure. However, the small sample size limits interpretability and stability, so the results below should be interpreted as exploratory indications of possible relationships, rather than definitive evidence of nonlinear or causal effects.

In terms of random forest results for TFV, aside from body weight and plasma TFV AUC, *Gemella*, *Megasphaera, Holdemanella*, and *Falsiporphyromonas* were the strongest microbial contributors (**Figure** **4** **c**). SHAP dependence plots suggested a generally positive association for *Gemella* and *Holdemanella*, whereas *Megasphaera* and *Falsiporphyromonas* tended to show negative associations (**Figure** **4** **d**). For 3TC, plasma 3TC AUC and age dominated predictions, followed by microbial genera including *Dialister*, *Prevotella*, and *Atopobium* (**Figure** **S9c**). SHAP dependence plots suggested potential curved patterns for several taxa, although these trends should be interpreted cautiously given the limited sample size (**Figure** **S9d**). For FTC, plasma FTC AUC and age emerged as the strongest clinical predictors in the RF model, followed by several microbial predictors (**Figure** **S10c**). Notably, *Bifidobacteriaceae* showed positive associations with cervical FTC‐TP exposure, and *Lachnospiraceae*, *Gardnerella*, and *Prevotellaceae* (not further classified to genus) tended to exhibit negative associations (**Figure** **S10d**).

XGBoost was also performed to identify influential predictors of antiretroviral drug exposures. For TFV, *Gemella*, *Falsiporphyromonas, Megasphaera, and Prevotella* were identified as key microbial predictors (**Figure** **4** **e,f**). For 3TC, plasma 3TC AUC and age again ranked highest in importance, followed by *Dialister*, *Prevotella*, *Atopobium*, and *Gardnerella* (**Figure** **S9e,f**). For FTC, besides plasma FTC AUC and weight, *Mycoplasmopsis*, *Lachnospiraceae*, *Bifidobacteriaceae*, and *Prevotellaceae* were identified as key microbial predictors (**Figure** **S10e,f**).

### Predictors selected by different machine learning methods

Across the three machine learning approaches (LASSO regression, Random Forest, and XGBoost), a consistent set of microbial and clinical predictors for cervical drug exposure was identified. For TFV, as expected, plasma TFV AUC, and body weight were selected as important predictors for TFV‐DP AUC in cervical tissues by all methods. Among microbial genera, *Gemella*, *Falsiporphyromonas*, and *Megasphaera* were repeatedly identified across all models (**Figure** **5**). For 3TC, plasma 3TC AUC, and age were consistently selected across all three methods. Among microbial genera, *Dialister*, *Prevotella*, *Atopobium, Streptobacillus*, and *Gardnerella* were repeatedly selected by at least two methods (**Figure** **S11**). For FTC, *Bifidobacteriaceae* was the only microbial predictor selected by all three methods. In addition, plasma FTC AUC, age, *Lachnospiraceae*, and *Prevotellaceae* were repeatedly selected by at least two approaches (**Figure** **S12**).

**Figure 5:**
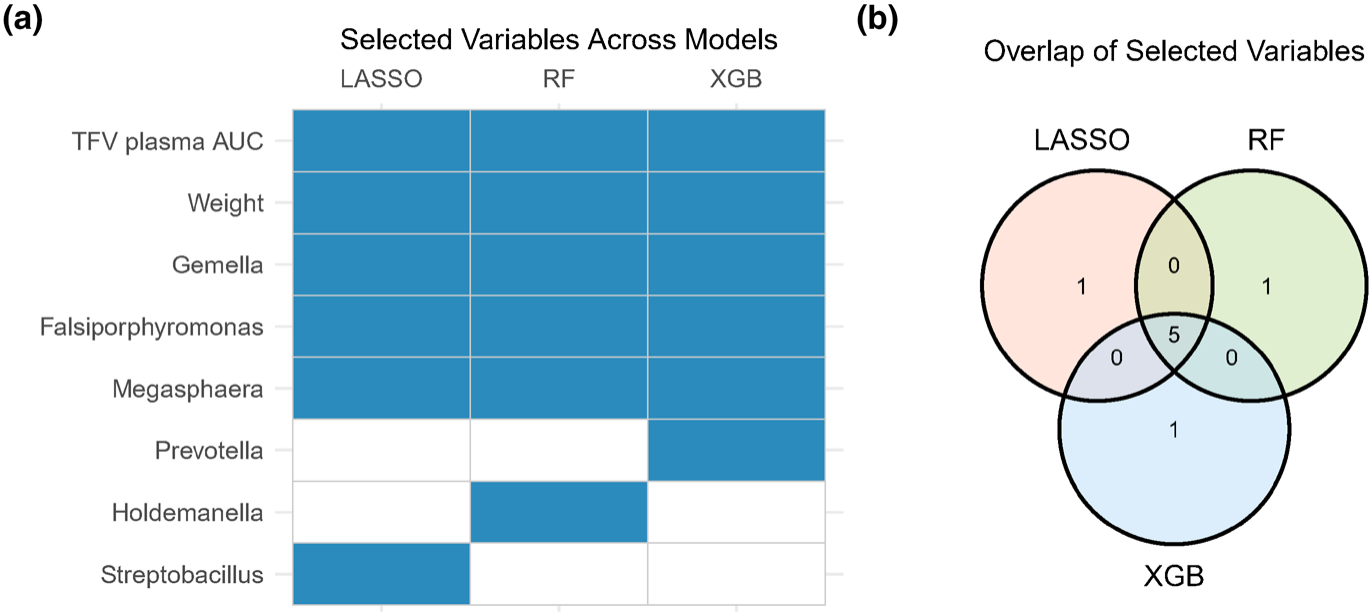
Predictors selected by different machine learning methods for TFV cervical exposure. (a) Heatmap summarizing the top‐ranked clinical and microbial predictors selected by LASSO regression, Random Forest (RF), and XGBoost (XGB). (b) Venn diagram showing the overlap of top‐ranked predictors across methods.

### Microbial Co‐occurrence network analysis

SparCC‐based co‐occurrence analysis revealed distinct microbial interaction patterns within the vaginal microbiome. Among Uganda women with TFV and 3TC data, positive correlations were mainly observed among *Prevotella, Sneathia, Dialister, Megasphaera, Atopobium*, and *Parvibacter*, while *Lactobacillus* showed negative correlations with these taxa, reflecting its mutual exclusivity with these genera (**Figure** **6**). Networks were not constructed for the US study due to low sample size, which is likely to produce inconsistent results.^36^

**Figure 6:**
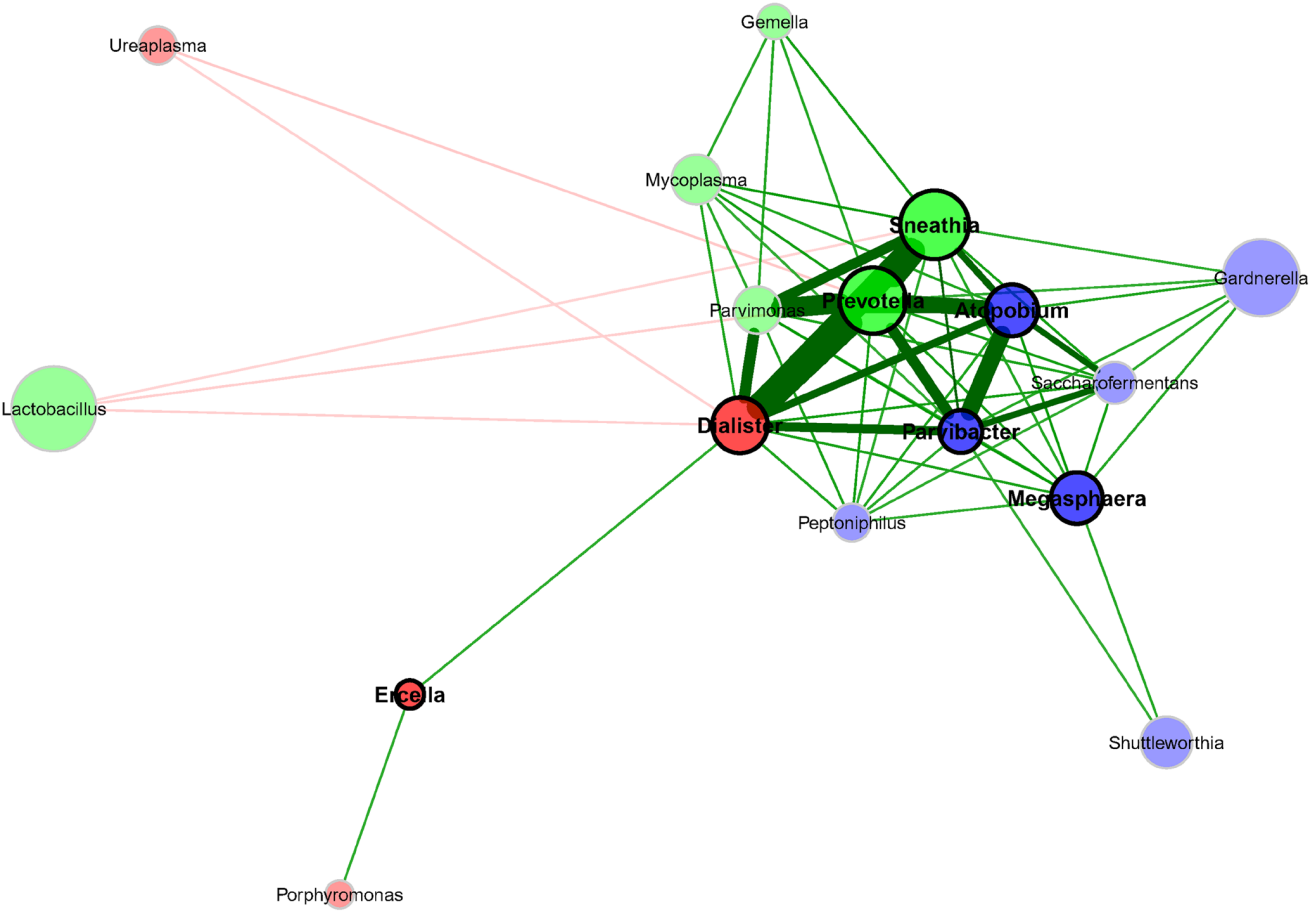
SparCC‐based co‐occurrence network of the vaginal microbiome in the Uganda study including TFV and 3TC data. Each node represents a genus, and edges indicate significant correlations (|*r*| ≥ 0.30). Positive correlations (green edges) represent taxa that tended to co‐occur, while negative correlations (red edges) indicated mutual exclusion.

## DISCUSSION

In this study, we integrated a PMx modeling approach with ML methods to investigate the impact of the vaginal microbiome on antiretroviral drug exposure in the female genital tract. *Gemella*, *Megasphaera*, and *Falsiporphyromonas* were consistently identified as the most robust microbial predictors of cervical TFV‐DP exposure across three ML approaches. In contrast, *Dialister*, *Prevotella*, *Atopobium, Streptobacillus*, and *Gardnerella* abundances were significantly associated with cervical 3TC‐TP exposure, whereas *Bifidobacteriaceae*, *Lachnospiraceae*, and *Prevotellaceae* were significantly associated with cervical FTC‐TP exposure. Overall, we placed the greatest emphasis on genera consistently identified across multiple ML methods, as these approaches prioritized robust signals in high‐dimensional microbiome data while treating the heatmap correlation and differential abundance analyses as supportive and exploratory. Notably, we did not observe differences in overall microbiome diversity, such as alpha diversity (species richness and/or evenness) or beta diversity (community dissimilarity) associated with differing drug exposures. Instead, specific taxon‐level associations between drug exposures and cervical tissues were identified.

In gastrointestinal pharmacomicrobiomics, it is well recognized that gut commensals can influence drug PK through several mechanisms, including direct drug metabolism and modulation of immune responses.^37^ While less diverse and abundant than the gut, it is anticipated that the vaginal microbiome can similarly influence mucosal drug exposure.^8^, ^10^, ^38^ Our findings support the emerging view that mucosal microbiota can modulate drug PK via direct and indirect mechanisms.^8^, ^10^

A secondary analysis of the CAPRISA 004 TFV gel study found that improved efficacy was observed in women with *Lactobacillus*‐dominant microbiomes compared to those with *Gardnerella*‐dominant anaerobic communities.^10^ Similarly, a study evaluating safety and PK of 6 daily applications of TFV vaginal gel or film showed that cervical tissue TFV‐DP levels were higher in women having a Nugent score 0–3 (reflecting *Lactobacillus* dominance) but were lower in those with greater *Gardnerella* abundance.^39^ *In vitro* studies have demonstrated that *Gardnerella* can deplete TFV by metabolism more rapidly than pharmacological activations of TFV to TFV‐DP by host target cells. A similar microbiome‐driven TFV depletion has also been observed with *Prevotella*, *Peptostreptococcus*, and *Mobiluncus*, and to a lesser extent with *Escherichia*, but not with *Lactobacillus*.^8^, ^10^ In our study, *Megasphaera* was identified as a robust negative predictor of cervical TFV‐DP AUC across multiple models. Microbial co‐occurrence network analysis further showed that *Megasphaera* strongly co‐occurred with *Gardnerella*, suggesting these taxa may reflect or contribute to vaginal community states associated with reduced mucosal drug concentrations. Additionally, McClelland *et al*. reported that *Megasphaera* was associated with a greater than 4.5‐fold increased risk of HIV acquisition, although its potential role in disrupting oral PrEP efficacy remains unknown.^40^

However, a clinical study among African women with a high prevalence of bacterial vaginosis (which is strongly associated with higher abundance of *Gardnerella* and other anaerobes) and high oral PrEP adherence found no difference in HIV prevention efficacy between women with abnormal versus normal vaginal microbiota.^41^ Another study found no significant associations between vaginal TFV concentrations and microbiota composition, or any predictors of discordance between blood and vaginal drug concentrations in women using daily oral PrEP. In some cases, women with *Gardnerella* were more likely to have higher TFV concentrations in cervicovaginal fluid.^42^ A possible explanation for the discrepancy between our findings and those from prior oral PrEP studies is that previous studies primarily relied on cervicovaginal fluid or systemic drug concentrations, which do not capture intracellular drug concentrations within tissue. In contrast, our study simulated cervical tissue TFV‐DP exposure by leveraging ectocervical biopsies and a PMx modeling approach, reflecting a more direct proxy of pharmacologic protection.

Differences between the Ugandan and US cohorts should also be considered when interpreting our findings. One important treatment difference was the use of a distinct nucleoside reverse transcriptase inhibitor (NRTI) backbone in the two cohorts. TFV is administered as a prodrug. While TDF was used as a prodrug in both cohorts, TAF was only used as a prodrug in a subset of the US cohort. TDF and TAF have distinct systemic and female genital tract PK profiles.^17^, ^18^ TAF is more stable in plasma and is converted intracellularly to TFV‐DP, resulting in substantially lower systemic TFV exposure than an equipotent dose of TDF.^18^, ^43^ Following a single dose, TFV‐DP concentrations were lower and frequently unquantifiable in female genital tract tissues after TAF compared with TDF.^44^ However, following multiple dosing, TFV‐DP concentrations measured 4 hours after the last dose were higher in female genital tract tissues among participants receiving TAF than among those receiving TDF.^18^ Given these distinct PK profiles, the TFV analysis was restricted to the Ugandan cohort, in which all participants received TDF. The NRTI backbones also differed with respect to 3TC and FTC: all Ugandan participants received 3TC, whereas all US participants received FTC. Accordingly, each drug was evaluated only in its respective cohort. Because each drug was analyzed within a single cohort, differences in NRTI backbones between the Ugandan and US cohorts would not directly confound the microbiome‐drug‐exposure associations identified for each drug. In addition, the third anchor drugs may represent an additional source of variability. In the Ugandan cohort, 49 of 50 participants received efavirenz, resulting in relatively homogenous anchor drug exposure. In contrast, the US study protocol did not specify a particular anchor drug, and treatment regimens were therefore varied across participants. Although drug–drug interactions among antiretroviral agents could alter systemic NRTI exposure,^45^, ^46^, ^47^ whether differences in anchor drugs meaningfully affect intracellular TFV, 3TC, or FTC exposure in the female genital tract or vaginal microbiome composition remains unclear. Moreover, vaginal microbiome composition and microbial co‐occurrence patterns may also vary across geographic regions and populations. Previous studies have observed differences in vaginal microbiome composition among different ethnic groups. For example, vaginal bacterial communities dominated by *Lactobacillus* species were more common in Asian and White women than in Hispanic and Black women, whereas Hispanic and Black women were more likely to have more diverse anaerobic communities.^48^, ^49^ Since each drug was evaluated within a single cohort, population differences in vaginal microbiome composition are unlikely to bias the observed microbiome‐drug‐exposure associations, but should be considered when interpreting the generalizability of our findings. From a clinical perspective, our findings have important implications for optimizing HIV PrEP in women. First, the identification of specific bacteria associated with suboptimal mucosal drug exposure suggests that vaginal microbiome profiling could be used to inform the personalization of PrEP strategies. More practically, these findings may also help inform the development of simple biomarkers or risk signatures for women at risk of reduced mucosal antiretroviral exposure. For instance, women with a high abundance of *Megasphaera* or *Falsiporphyromonas* may be at risk for reduced cervicovaginal TFV‐DP concentrations, diminishing protective efficacy. Clinicians might consider more intensive adherence counseling, higher dosage or dosing frequency for such individuals. Notably, our simulations of the 2‐1‐1 on‐demand PrEP regimen showed that drug exposures in cervicovaginal tissue can be highly variable across individuals. A Recent study using the same PK/PD model has similarly explored alternative on‐demand dosing strategies in women, further highlighting the importance of optimizing PrEP regimens to achieve sufficient mucosal protection.^50^, ^51^ When the vaginal microbiome reduces TFV‐DP concentrations, achieving consistent mucosal protection with on‐demand dosing regimens may be difficult. This supports a cautious approach to implementing event‐driven PrEP in women with vaginal dysbiosis.^4^, ^5^ Importantly, our findings highlight the potential to integrate microbiome taxa into PMx frameworks for model‐informed precision dosing (MIPD).^52^ While PMx approaches frequently use covariate modeling based on predefined biological knowledge (such as full fixed^53^/random^54^ effect modeling), the interactions between specific microbiome genera and drug disposition remain largely unknown. While our previous study made early attempts to incorporate microbiome variables in PMx models,^55^ this persistent knowledge gap has hindered the broader integration of microbiome data into frameworks for MIPD. The present study bridges the gap by conducting an extensive screen of microbiome‐drug‐exposure associations for antiretroviral drugs. We identified key microbial predictors such as *Megasphaera*, *Falsiporphyromonas*, *Gemella*, and *Gardnerella*, which could be incorporated as covariates in future population PK models to refine individual‐level predictions of mucosal drug exposure. This would enable more accurate identification of female subgroups who may be at risk to fail HIV PrEP regimens and inform microbiome‐informed dosing strategies to optimize efficacy at the mucosal site.

A major strength of this study lies in the integration of a quantitative PMx model with data‐driven ML analyses to unravel microbiome‐drug exposure relationships. This generalizable PMx‐ML approach offers several methodological and translational advantages. First, model‐simulated cervical tissue exposures based on EBEs provide a more physiologically relevant measure of local drug exposure than single time‐point plasma concentrations. This approach captures concentration changes over time at the mucosal site of HIV acquisition, which is more relevant for evaluating PrEP protection. Although vaginal and cervical tissues represent distinct PK compartments and cervical concentrations may not directly reflect vaginal concentrations,^19^ they are anatomically connected mucosal sites within the female genital tract, and vaginal and cervical microbiome compositions have been reported to be closely related.^56^ Therefore, evaluating vaginal microbiome associations with cervical drug exposure remains biologically relevant. Second, the simulation capabilities of the PMx model enabled the evaluation of alternative dosing regimens *in silico*. This allowed us to link our findings to real‐world scenarios that were not directly observed in the clinical data. By simulating how different regimens affect mucosal exposure, we can evaluate whether specific microbiome compositions would associate with various drug concentrations under a unified dosing regimen. Third, integrating the PK model outputs with ML allows us to handle the high‐dimensional microbiome data in a robust manner. The use of multiple ML algorithms minimizes the risk of model‐specific bias, and the consistency of microbial predictors across methods increases confidence in their biological relevance. This framework is generalizable to other drug classes and mucosal tissues and offers a pathway toward MIPD that incorporates microbial covariates.

Despite its strengths, this study has several limitations. First, this was a retrospective secondary analysis of two completed clinical studies, and no prospective sample size or power calculation was performed. Consequently, the modest sample size and observational design may limit statistical power and generalizability. While the consistency of findings across multiple ML models provides confidence in the robustness of key associations, external validation in larger and more diverse cohorts is warranted. Second, although the population PK model was appropriately adapted for our analysis, it was not originally developed from the study population. Differences in study population, disease status, and dosing conditions may introduce bias due to potential deviations from the original model assumptions or parameter distributions. Importantly, the mechanistic role of specific microbial taxa in modulating mucosal drug exposure was inferred from statistical associations in observational clinical studies rather than direct experimental evidence. Unmeasured or uncontrolled host microbial variables, behavioral, or clinical factors may influence both vaginal microbiome composition and antiretroviral PK, resulting in residual confounding. Therefore, the identified associations should not be interpreted as causal, and experimental validations are essential to validate these microbiome‐drug interactions and to establish causality before clinical translation. In addition, because the present analyses were conducted primarily at the genus level, future evaluation of the most consistently identified taxa at the species level may help refine these association signals.

In summary, our study implemented an integrated PMx‐ML framework to evaluate the impact of the vaginal microbiome on antiretroviral drug exposure in female genital tract tissue. While overall microbiome diversity was not associated with drug exposure, specific taxa demonstrated significant associations. These findings may help guide future development of practical microbiome‐based biomarkers, inform MIPD, and support the optimization of PrEP strategies for women.

## FUNDING

This research was funded by Clinical and Translational Science Institute (CTSI) Office of Discovery and Translation (ODAT) Translational Science Funding Program.

## CONFLICT OF INTEREST

The authors declared no competing interests for this work.

## Author contributions

DY, LBC, CS, PJ, MN, and SC wrote the manuscript; CS, PJ, MN, and SC designed the research; DY, LBC, CS, FKM, MN, and SC performed the research; DY and SC analyzed the data.

## PREVIOUS PRESENTATIONS

A part of this work was presented in a poster at American Conference of Pharmacometrics (ACOP) 162025, Denver, CO.

## Supporting information


Data S1.

Data S2.

Data S3.
