# Supplementary material for: Evaluating Vaginal Microbiome Effects on Antiretroviral Exposure in the Female Genital Tract Using Pharmacometric and Machine Learning Approaches
Source: Clin Pharmacol Ther. 2026 Oct 3:10.1002/cpt.70499. Online ahead of print. doi: 10.1002/cpt.70499 (PMC13633485; doi:10.1002/cpt.70499)
Supplement: Supplementary file 1 [file CPT-9999-0-s001.docx]

**SUPPLEMENTAL METHODS**

**Data**

The first study is a PK substudy (NCT03377608) conducted in Kampala, Uganda between November 2017 and March 2018, which assessed the effect of depot medroxyprogesterone acetate (DMPA) contraceptive method on tenofovir diphosphate (TFV-DP) and lamivudine triphosphate (3TC-TP) cervical concentrations. The detailed study population and design have been described in our previous publication^1^. Briefly, 50 premenopausal women living with HIV, stable on their TDF/3TC regimen, and virally suppressed (plasma HIV RNA <50 copies/mL) for ≥6 months were enrolled. At a single visit, TFV and 3TC concentrations were quantified in plasma, and TFV-DP and 3TC-TP were quantified in ectocervical biopsies. Vaginal microbiome profiles were characterized by performing 16S ribosomal (r) RNA gene sequencing on DNA extracted from vaginal swabs collected at the same visit.

The second study, an observational PK study (NCT03218085), conducted in the United States between July 2017 and February 2024, examined the role of menopause and exogenous hormones on antiretroviral drug exposures^2^. Briefly, 21 pre- and post-menopausal women aged ≥18 years living with HIV, were stable on antiretroviral regimens containing TDF/TAF and FTC, and virally suppressed for ≥6 months were enrolled. At a single visit, TFV and FTC concentrations were quantified in plasma, and TFV-DP and emtricitabine triphosphate (FTC-TP) concentrations were quantified in cervical tissue. Vaginal microbiome abundances were acquired using both 16S rRNA and shotgun sequencing, with the latter available for 13 of 21 subjects.

Sequence data were processed using mothur (version 1.41.1) and [the bioinformatics pipeline described previously](https://pubmed.ncbi.nlm.nih.gov/31131846/)^1^. The resulting taxonomic abundance tables were imported into R and handled using the phyloseq package for downstream analyses. Downstream microbiome analyses were conducted primarily at the genus level; however, for taxa that could not be reliably resolved to the genus level, the lowest available taxonomic level (e.g., family level) was retained. A total of 181 genera were identified in the Uganda dataset, with a median of 25 detected genera per participant. In the U.S. dataset, 196 genus/family-level features were identified, with a median of 45 detected features per participant (Table S4).

**Model-Based Estimation and Simulation of Antiretroviral Exposure**

Deterministic simulations using derived EBEs were performed using mrgsolve. Random effects were set to zero to ensure simulated concentration-time profiles were driven by EBEs only, without random sampling. Simulations of the two multi-dose regimens described above were initialized at time 0 (first dose) and conducted over 0–240 h using a time grid of 0.1 h. The 240-h duration was simulated to capture the full simulated dosing period, including the extended 2-1-1-1-1-1 regimen, and to assess overall exposure and protection profiles after the initial dose.

**Ex-Vivo Determination of TFV-DP EC_50_**

A cervical explant model was used to estimate the EC_50_ of TFV-DP against HIV. Cervical explants are established as previously described^3^. Tissue explants (n=38) were incubated at drug concentrations 0, 100, or 300 μg/mL in a 48-well plate for 24 hours, then incubated for three hours with HIV-1_JR-CSF_ in a 48-well tissue culture plate. One uninfected explant per donor was collected for TFV-DP quantification. Explants were then rinsed and transferred to prepared gelfoam raft constructs. The rafts were hydrated in culture media containing antiretroviral at concentrations kept constant throughout the entire culture period. HIV RNA was measured via real-time PCR in explants collected on Days 1, 2, and 4 (RNEasy, Qiagen). TFV-DP EC_50_ of 10.7 (standard error 10.6) fmol/mg was calculated using PROC NLIN in SAS 9.4 (Cary, NC).

**Association Analyses between Drug Exposures and Microbiome**

*Differential Abundance Analysis*

Considering the limited sample size of our clinical study, statistical significance was assessed with more liberty using the Wald test with false discovery rate correction (FDR) < 0.2 considering the exploratory nature of the analysis.

*Machine Learning Models*

Regression-based Modeling with Ridge and LASSO

Microbiome data are highly dimensional and exhibit substantial multicollinearity, conditions under which ordinary least squares (OLS) regression performs poorly. Ridge regression is a well-established method for addressing multicollinearity by applying *L₂* regularization, which adds a penalty proportional to the squared magnitude of regression coefficients. This penalization shrinks coefficient estimates toward zero, reducing model variance and improving robustness to overfitting at the cost of introducing slight bias^4^. LASSO regression employs *L₁* regularization, which penalizes the absolute values of regression coefficients, driving coefficients of less important variables to exactly zero and thereby performing automatic variable selection^5^. Compared with ridge regression, LASSO yields a more interpretable model by retaining only the most predictive features. Regularization parameters (i.e., hyperparameters) for both ridge and LASSO models were optimized using leave-one-out cross-validation (LOOCV).

Tree-based Modeling with Random Forest and XGBoost

Recently, RF and XGBoost have been increasingly used in the fields of computational biology and pharmacometrics. Microbiome data inherently exhibit high-dimensionality, sparsity and compositional structure, and frequently have small sample sizes. These properties are well handled by tree-based method such as RF and XGBoost, thereby these methods are deemed appropriate for analyzing microbiome data in our study^6,7^. RF is an ensemble learning method that constructs several randomized decision trees using bootstrap samples of the data and aggregates their predictions by averaging to introduce randomness and reduce overfitting^8^. RF regression models were developed using the *randomForest* package (version 4.7.1.2). Gradient-boosted tree models were implemented through the XGBoost framework using the *xgboost* package (version 1.7.11.1), which iteratively builds decision trees that correct residual errors from previous iterations and incorporates regularization to control model complexity^9,10^. Hyperparameters including maximum tree depth, learning rate, minimum number of observations at each leaf, and *L_1_*/*L_2_* penalties were optimized through 5-fold cross-validation with early stopping. To enable model interpretability, SHapley Additive exPlanations (SHAP) values were computed using the *fastshap* (version 0.1.1) and *SHAPforxgboost* (version 0.1.3) packages to estimate the marginal contribution of each microbiome genera or clinical covariates to the predicted cervical drug exposure^11^. Both SHAP summary (beeswarm) plots and SHAP dependence plots (top 6 features) were generated to characterize effect direction and relationships.

**Microbial Co-occurrence Network Analysis**

Genus-level microbiome abundance data were imported into R and combined with sample metadata to create a phyloseq object using the *phyloseq* package (version 1.46.0), which served as the input for downstream network analysis. Microbial associations were inferred using SparCC correlations implemented via the netConstruct function in the *NetCoMi* package (version 1.2.0) to account for the compositional nature of microbiome data. The resulting SparCC correlation matrix was thresholded at an absolute correlation coefficient of ≥0.30 to define an undirected adjacency matrix in which nodes represented microbial genera and edges indicated significant co-occurrence relationships. Network communities were identified using the fast-greedy clustering algorithm. Hub taxa, which is potential keystone genera driving network organization, were identified based on degree, betweenness, and closeness centrality metrics, with hubs defined as those in the top 10th percentile (threshold = 0.9) of each metric. Network construction and analysis followed approaches described previously^12^.

References:

1.	Nicol MR, Eneh P, Nakalega R, et al. Depot Medroxyprogesterone Acetate and the Vaginal Microbiome as Modifiers of Tenofovir Diphosphate and Lamivudine Triphosphate Concentrations in the Female Genital Tract of Ugandan Women: Implications for Tenofovir Disoproxil Fumarate/Lamivudine in Preexposure Prophylaxis. *Clin Infect Dis*. 2019;70(8):1717-1724. doi:10.1093/cid/ciz443

2.	Role of Exogenous and Endogenous Sex Hormones on Tenofovir and Emtricitabine. National Library of Medicine (US). <https://clinicaltrials.gov/study/NCT03218085>

3.	Collins LB, Le A, Nicol MR. Pharmacological evaluation of an ex vivo cervicovaginal HIV prevention model. *J Antimicrob Chemother*. 2026;81(4)doi:10.1093/jac/dkag103

4.	McDonald GC. Ridge regression. *WIREs Computational Statistics*. 2009;1(1):93-100. doi:<https://doi.org/10.1002/wics.14>

5.	Tibshirani R. Regression Shrinkage and Selection Via the Lasso. *Journal of the Royal Statistical Society: Series B (Methodological)*. 2018;58(1):267-288. doi:10.1111/j.2517-6161.1996.tb02080.x

6.	Qi Y. Random forest for bioinformatics. *Ensemble machine learning*. Springer; 2012:307-323.

7.	Wiens M, Verone‐Boyle A, Henscheid N, Podichetty JT, Burton J. A tutorial and use case example of the eXtreme gradient boosting (XGBoost) artificial intelligence algorithm for drug development applications. *Clin Transl Sci*. 2025;18(3):e70172.

8.	Breiman L. Random forests. *Machine learning*. 2001;45(1):5-32.

9.	Chen T. XGBoost: A Scalable Tree Boosting System. *Cornell University*. 2016;

10.	Chen T, He T, Benesty M, Khotilovich V. Package ‘xgboost’. *R version*. 2019;90(1-66):40.

11.	Mosca E, Szigeti F, Tragianni S, Gallagher D, Groh G. SHAP-based explanation methods: a review for NLP interpretability. 2022:4593-4603.

12.	Ziegert Zachary A, Troester A, Frebault J, et al. SparCC co-occurrence networking reveals intracommunity dynamics of the microbiome following colorectal surgery. *Microbiology Spectrum*. 2026;0(0):e03973-25. doi:10.1128/spectrum.03973-25
