# Supplementary material for: Evaluating Vaginal Microbiome Effects on Antiretroviral Exposure in the Female Genital Tract Using Pharmacometric and Machine Learning Approaches
Source: Clin Pharmacol Ther. 2026 Oct 3:10.1002/cpt.70499. Online ahead of print. doi: 10.1002/cpt.70499 (PMC13633485; doi:10.1002/cpt.70499)
Supplement: Supplementary file 2 [file CPT-9999-0-s003.docx]

**Figure S1. Diagram for TFV PK model developed by Leung, E. *et al^1^*.** The model incorporates several key components: oral absorption through seven gut transit compartments from the depot, two-compartment plasma disposition, and distribution to three mucosal tissue sites (vaginal, cervical, and rectal). Each tissue contains both parent drug (TFV/FTC) and active metabolite (tenofovir diphosphate [TFV-DP]/emtricitabine triphosphate [FTC-TP]) compartments to describe intracellular phosphorylation. Original model was developed using data available for compartments outlined by red. We have TFV data available for compartments filled in yellow. Ka: absorption rate constant (/hour); Vc: central volume of distribution (L); CLtt: total drug clearance (L/hour); CLvvtp: clearance of metabolite from vaginal compartment (L/hour); Clvetp: clearance of metabolite from cervical compartment (L/hour); Kg: gut transit rate (/hour).

**Figure S2.** **Derive empirical Bayes estimates (EBEs) to drive simulations for 3TC.** (a) Predictive checks using population PK model developed by Leung, E. *et al*. The dots are observed concentrations, with red triangle represents observations below limit of quantification. The shaded areas are concentrations simulated repetitively by model (n=200), with transparency represents median, 50%, 80%, and 90% prediction intervals of the simulations. 3TC: lamivudine; 3TC-TP: lamivudine triphosphate. (b) Empirical Bayes estimates (EBEs) derived using Maximum A Posterior (MAP) Bayesian method. The histograms represent derived EBE based on the observed plasma and cervical tissue concentration-time data. Shaded areas represented the theoretical distributions of the parameters*.* IIV: inter-individual variability; SHK: shrinkage; Ka: absorption rate constant (/hour); Vc: central volume of distribution (L); CLttvvtp: clearance rate of drug from vaginal compartment (L/hour); CLvvtp: clearance rate of metabolite from vaginal compartment (L/hour); CLttve: clearance rate of drug from cervical compartment (L/hour); Clvetp: clearance rate of metabolite from cervical compartment (L/hour); CLttvr: clearance rate of drug from rectal compartment (L/hour); Kg: gut transit rate (/hour).

**Figure S3.** **Derive empirical Bayes estimates (EBEs) to drive simulations for FTC.** (a) Predictive checks using population PK model developed by Leung, E. *et al*. The dots are observed concentrations, with red triangle represents observations below limit of quantification. The shaded areas are concentrations simulated repetitively by model (n=200), with transparency represents median, 50%, 80%, and 90% prediction intervals of the simulations. FTC: emtricitabine; FTC-TP: emtricitabine triphosphate. (b) Empirical Bayes estimates (EBEs) derived using Maximum A Posterior (MAP) Bayesian method. The histograms represent derived EBE based on the observed plasma and cervical tissue concentration-time data. Shaded areas represented the theoretical distributions of the parameters*.* IIV: inter-individual variability; SHK: shrinkage; Ka: absorption rate constant (/hour); Vc: central volume of distribution (L); CLttvvtp: clearance rate of drug from vaginal compartment (L/hour); CLvvtp: clearance rate of metabolite from vaginal compartment (L/hour); CLttve: clearance rate of drug from cervical compartment (L/hour); Clvetp: clearance rate of metabolite from cervical compartment (L/hour); CLttvr: clearance rate of drug from rectal compartment (L/hour); Kg: gut transit rate (/hour).

**Figure S4. Association between overall vaginal microbiome diversity and TFV cervical tissue exposures.** TFV cervical exposure groups were dichotomized as high or low based on the median simulated TFV-DP AUC in cervical tissues. (a) The Shannon index and (b) Chao1 index of alpha diversity by TFV cervical exposure group. (c) Principal coordinate analysis (PCoA) of beta diversity by TFV cervical exposure group.

**Figure S5. Association between overall vaginal microbiome diversity and 3TC cervical tissue exposures.** 3TC cervical exposure groups were dichotomized as high or low based on the median simulated 3TC-TP AUC in cervical tissues. (a) The Shannon index and (b) Chao1 index of alpha diversity by 3TC cervical exposure group. (c) Principal coordinate analysis (PCoA) of beta diversity by 3TC cervical exposure group.

**Figure S6. Association between overall vaginal microbiome diversity and FTC cervical tissue exposures.** FTC cervical exposure groups were dichotomized as high or low based on the median simulated FTC-TP AUC in cervical tissues. (a) The Shannon index and (b) Chao1 index of alpha diversity by FTC cervical exposure group. (c) Principal coordinate analysis (PCoA) of beta diversity by FTC cervical exposure group.

**Figure S7. Associations between vaginal microbiome composition and cervical 3TC exposure.** (a) Heatmap showing the associations between CLR-transformed microbiome relative abundance and simulated drug exposure metrics. Top color bar represents the microbiome phylum. AUC_P: plasma 3TC AUC; AUC_T: cervical tissue 3TC-TP AUC; CMAX_P: plasma 3TC peak concentration; CMAX_T: cervical tissue 3TC-TP peak concentration; CAVE_P: plasma 3TC average concentration; CAVE_T: cervical tissue 3TC-TP average concentration. (b) Volcano plot showing differential abundance analysis comparing high versus low cervical 3TC-TP AUC groups. The horizontal dashed line indicates the false discovery rate (FDR) threshold (0.2), and vertical dashed lines denote log2 fold change cutoffs. Genera meeting the FDR threshold are highlighted and labeled, with red indicating enrichment in the high-exposure group and blue indicating enrichment in the low-exposure group.

**Figure S8. Associations between vaginal microbiome composition and cervical FTC exposure.** (a) Heatmap showing the associations between CLR-transformed microbiome relative abundance and simulated drug exposure metrics. Top color bar represents the microbiome phylum. AUC_P: plasma FTC AUC; AUC_T: cervical tissue FTC-TP AUC; FRAC_1: fraction of time above threshold 1 (FTC-TP:dCTP=0.27, TZM-bl cell)^2^; FRAC_2: fraction of time above threshold 2 (FTC-TP:dCTP=0.07, CD4+ T cell)^2^. (b) Volcano plot showing differential abundance analysis comparing high versus low cervical FTC-TP AUC groups. The horizontal dashed line indicates the false discovery rate (FDR) threshold (0.2), and vertical dashed lines denote log2 fold change cutoffs. Genera meeting the FDR threshold are highlighted and labeled, with red indicating enrichment in the high-exposure group and blue indicating enrichment in the low-exposure group.

**Figure S9. Machine learning-based analyses of predictors associated with 3TC-TP cervical exposure.** (a) Standardized coefficients from the LASSO regression model for 3TC-TP cervical tissue AUC. (b) Scatterplots of 3TC-TP cervical tissue AUC vs. LASSO-screened predictors. (c) SHAP summary plot from the random forest model, ranking predictors by their overall contribution to model predictions. (d) SHAP dependence plots from the random forest model for top-ranked predictors. (e) SHAP summary plot from XGBoost model, ranking predictors by their overall contribution to model predictions. (f) SHAP dependence plots from XGBoost model for top-ranked predictors. For SHAP summary plots, each point represents one observation for a predictor. The x-axis shows the SHAP value, indicating the direction and magnitude of that predictor’s contribution to the predicted 3TC-TP cervical tissue AUC; positive values increase the prediction, whereas negative values decrease it. Colors indicate relative predictor values from low to high, as shown in the color scale. X3tc_auc_p_d1: 3TC plasma AUC under 2-1-1 dosing regimen.

**Figure S10. Machine learning-based analyses of predictors associated with FTC-TP cervical exposure.** (a) Standardized coefficients from the LASSO regression model for FTC-TP cervical tissue AUC. (b) Scatterplots of FTC-TP cervical tissue AUC vs. LASSO-screened predictors. (c) SHAP summary plot from the random forest model, ranking predictors by their overall contribution to model predictions. (d) SHAP dependence plots from the random forest model for top-ranked predictors. (e) SHAP summary plot from XGBoost model, ranking predictors by their overall contribution to model predictions. (f) SHAP dependence plots from XGBoost model for top-ranked predictors. For SHAP summary plots, each point represents one observation for a predictor. The x-axis shows the SHAP value, indicating the direction and magnitude of that predictor’s contribution to the predicted FTC-TP cervical tissue AUC; positive values increase the prediction, whereas negative values decrease it. Colors indicate relative predictor values from low to high, as shown in the color scale. Ftc_auc_p_d1: FTC plasma AUC under 2-1-1 dosing regimen.

**Figure S11. Predictors selected by different machine learning methods for 3TC cervical exposure.** (a) Heatmap summarizing the top-ranked clinical and microbial predictors selected by LASSO regression, Random Forest (RF), and XGBoost (XGB). (b) Venn diagram showing the overlap of top-ranked predictors across methods.

**Figure S12. Predictors selected by different machine learning methods for FTC cervical exposure.** (a) Heatmap summarizing the top-ranked clinical and microbial predictors selected by LASSO regression, Random Forest (RF), and XGBoost (XGB). (b) Venn diagram showing the overlap of top-ranked predictors across methods.

**Table S1. Summary of (a) exposure metrics and (b) fraction of time above thresholds under 2-1-1 dose of TFV**

(a)

| Exposures | Plasma (TFV) | Cervical Tissue (TFV-DP) |
| --- | --- | --- |
| AUC_0-240_ | 8.95 × 10³ (1.64 × 10³) ng·h/mL | 1.35 × 10⁶ (1.20 × 10⁶) fmol·h/mL |
| C_max_ | 368 (69.7) ng/mL | 1.41 × 10⁴ (9.11 × 10³) fmol/mL |
| C_ave_ | 37.3 (6.84) ng/mL | 5.63 × 10³ (4.99 × 10³) fmol/mL |

(b)

| TFV-DP fraction of time above thresholds in cervical tissues | Threshold 1 | Threshold 2 | Threshold 3 |
| --- | --- | --- | --- |
| Within 240 hours after first dose | 0.282 (0.221) | 0.0321 (0.105) | 0.192 (0.227) |
| Within 24 hours after last dose | 0.708 (0.426) | 0.102 (0.294) | 0.481 (0.480) |

Data are shown as mean (sd). Threshold 1: TFV-DP:dATP ratio of 0.086 (EC_90_ in TZM-bl cell)^2^; Threshold 2: TFV-DP:dATP ratio of 0.29 (EC_90_ in CD4+ T cell)^2^; Threshold 3: TFV-DP concentration of 10,700 fmol/g (cervical explant EC₅₀). TFV: tenofovir; TFV-DP: tenofovir diphosphate; dATP: deoxyadenosine triphosphate.

**Table S2. Summary of exposure metrics under 2-1-1 dose of 3TC**

| Exposures | Plasma (3TC) | Cervical Tissue (3TC-TP) |
| --- | --- | --- |
| AUC_0-240_ | 5.66 × 10⁴ (801) ng·h/mL | 2.59 × 10⁷ (1.60 × 10⁷) fmol·h/mL |
| C_max_ | 3.60 × 10³ (612) ng/mL | 5.30 × 10⁵ (3.08 × 10⁵) fmol/mL |
| C_ave_ | 236 (3.34) ng/mL | 1.08 × 10⁵ (6.66 × 10⁴) fmol/mL |

Data are shown as mean (sd).

**Table S3. Summary of (a) exposure metrics and (b) fraction of time above thresholds under 2-1-1 dose of FTC**

(a)

| Exposures | Plasma (FTC) | Cervical Tissue (FTC-TP) |
| --- | --- | --- |
| AUC_0-240_ | 3.80 × 10⁴ (737) ng·h/mL | 1.73 × 10⁷ (1.30 × 10⁷) fmol·h/mL |
| C_max_ | 2.55 × 10³ (348) ng/mL | 3.62 × 10⁵ (2.58 × 10⁵) fmol/mL |
| C_ave_ | 158 (3.07) ng/mL | 7.22 × 10⁴ (5.43 × 10⁴) fmol/mL |

(b)

| FTC-TP fraction of time above thresholds in cervical tissues | Threshold 1 | Threshold 2 |
| --- | --- | --- |
| Within 240 hours after first dose | 0.504 (0.0787) | 0.655 (0.0842) |
| Within 24 hours after last dose | 1.00 (0.00) | 1.00 (0.00) |

Data are shown as mean (sd). Threshold 1: FTC-TP:dCTP ratios of 0.27 (EC_90_ in TZM-bl cell)^2^; Threshold 2: FTC-TP:dCTP ratios of 0.07 (EC_90_ in CD4+ T cell)^2^. FTC: emtricitabine; FTC-TP: emtricitabine triphosphate; dCTP: deoxycytidine triphosphate.

References:

1.	Leung E, Cottrell ML, Sykes C, White N, Kashuba ADM, Dumond JB. A multicompartment population PK model to predict tenofovir and emtricitabine mucosal tissue concentrations for HIV prevention. *CPT Pharmacometrics Syst Pharmacol*. Dec 2023;12(12):1922-1930. doi:10.1002/psp4.13042

2.	Cottrell ML, Yang KH, Prince HM, et al. A Translational Pharmacology Approach to Predicting Outcomes of Preexposure Prophylaxis Against HIV in Men and Women Using Tenofovir Disoproxil Fumarate With or Without Emtricitabine. *J Infect Dis*. Jul 1 2016;214(1):55-64. doi:10.1093/infdis/jiw077
